# Long-term efficacy, safety, and immunogenicity of the adalimumab biosimilar, PF-06410293, in patients with rheumatoid arthritis after switching from reference adalimumab (Humira®) or continuing biosimilar therapy: week 52–92 data from a randomized, double-blind, phase 3 trial

**DOI:** 10.1186/s13075-021-02626-4

**Published:** 2021-09-25

**Authors:** Roy M. Fleischmann, Daniel F. Alvarez, Amy E. Bock, Carol Cronenberger, Ivana Vranic, Wuyan Zhang, Rieke Alten

**Affiliations:** 1 University of Texas Southwestern Medical Center at Dallas, Metroplex Clinical Research Center, Dallas, TX USA; 2 Pfizer Inc., Collegeville, PA USA; 3 Pfizer Inc., Cambridge, MA USA; 4 Pfizer, Tadworth, UK; 5 Pfizer Inc., Lake Forest, IL USA; 6 Schlosspark-Klinik, University Medicine Berlin, Berlin, Germany

**Keywords:** Adalimumab, Adalimumab-afzb, Biosimilar, PF-06410293, Rheumatoid arthritis, Long-term, Switch, Safety

## Abstract

**Background/objective:**

REFLECTIONS B538–02 is a randomized, double-blind comparative study of the adalimumab (ADL) biosimilar PF-06410293, (ADL-PF), and reference ADL sourced from the European Union (ADL-EU) in patients with active RA. Therapeutic equivalence was demonstrated based on ACR20 responses at week 12 (primary endpoint). We report long-term safety, immunogenicity, and efficacy of ADL-PF in patients who continued ADL-PF treatment throughout 78 weeks or who switched from ADL-EU to ADL-PF at week 26 or week 52.

**Methods:**

Eligible patients (2010 ACR/EULAR RA diagnosis criteria for ≥ 4 months; inadequate response to MTX, ≤ 2 doses non-ADL biologic), stratified by geographic regions were initially randomized (1:1) in treatment period 1 (TP1) to ADL-PF or ADL-EU (40 mg subcutaneously, biweekly), both with MTX (10–25 mg/week). At week 26 (start of TP2), patients receiving ADL-EU were re-randomized to remain on ADL-EU or transition to ADL-PF for 26 weeks. At week 52 (start of TP3), all patients received open-label treatment with ADL-PF for 26 weeks and were followed after last treatment dose to week 92. To evaluate maintenance of response after switching or remaining on ADL-PF, ACR20, DAS28-4(CRP), and other measures of clinical response/remission were assessed through week 78 as secondary endpoints. Three groups were evaluated: biosimilar, week 26 switch, and week 52 switch.

**Results:**

Overall, 507 patients participated in TP3. ACR20 response rates at week 52 were 88.4%, 88.2%, and 87.6% for the biosimilar, week 26, and week 52 switch groups, respectively. ACR20 response rates and DAS28-4(CRP) scores were sustained and comparable across groups in TP3. Incidence of treatment-emergent adverse events (AEs) during TP3 and follow-up was 42.6% (biosimilar), 37.0% (week 26 switch), and 50.8% (week 52 switch); 3 (0.6%) patients (all week 52 switch) reported treatment-related serious AEs. ADL-PF was generally well tolerated, with a comparable safety profile across groups. Overall, incidences of patients with anti-drug antibodies in TP3 and follow-up were comparable among groups (46.1%, 46.5%, and 54.2%, respectively).

**Conclusions:**

There were no clinically meaningful differences in safety, immunogenicity, and efficacy for patients who were maintained on ADL-PF for 78 weeks and those who had switched from ADL-EU at week 26 or week 52.

**Trial registration:**

ClinicalTrials.gov, NCT02480153. First posted on June 24, 2015; EU Clinical Trials Register; EudraCT number: 2014-000352-29. Start date, October 27, 2014

**Supplementary Information:**

The online version contains supplementary material available at 10.1186/s13075-021-02626-4.

## Introduction

Rheumatoid arthritis (RA) is a lifelong chronic disease, which, although not curable, can be brought into remission or low disease activity in many patients by appropriate treatment [1]. Disease-modifying anti-rheumatic drugs (DMARDs) are effective treatments for RA, with the biologic DMARD adalimumab (ADL) being a well-established treatment for patients with this disease, as well as for patients with other inflammatory and autoimmune conditions [2, 3].

ADL is a recombinant, fully human, immunoglobulin G1 monoclonal antibody that specifically binds to human tumor necrosis factor-α (TNF-α), inhibiting its interaction with surface TNF receptors. Inhibition of TNF-α results in the down-regulation of the abnormal inflammatory pathways implicated in the pathogenesis and progression of immune-mediated inflammatory diseases [2, 3]. In clinical trials, ADL in combination with methotrexate has been shown to reduce clinical symptoms of RA, inhibit the progression of structural joint damage, improve functional capacity, reduce disability, and affect clinical remission [4–7]. Despite their efficacy, the timely and widespread use of biologic original DMARDs (boDMARDs) may be limited by high drug costs, resulting in difficult access for many patients and inequity of care across countries [1]. Biosimilars, which are biologic agents that are structurally highly similar, but not identical, and functionally equivalent [8, 9], to the approved reference product [10, 11], provide the opportunity to improve access to treatment by enabling cost savings to healthcare budgets, assuming their price is low and their use is reinforced by payors and health authorities, and accepted by patients [1].

PF-06410293 (ADL-PF) is an ADL biosimilar DMARD (bsDMARD), approved in the US (adalimumab-afzb; Pfizer Inc., New York, NY, USA) and the EU (Pfizer Europe MA EEIG, Brussels, Belgium) for all eligible indications of reference ADL, the boDMARD (Humira®; AbbVie Inc., North Chicago, IL, USA, and AbbVie Deutschland GmbH Co. KG, Ludwigshafen, Germany) [12]. Preclinical studies have shown ADL-PF to have a primary amino acid sequence identical to that of reference ADL and to have a similar profile in comparative analytical (structural and functional) and toxicology assessments [13]. Pharmacokinetic (PK) similarity of ADL-PF to both EU- and US-sourced reference ADL (ADL-EU and ADL-US, respectively) was demonstrated in healthy subjects [12]. In a randomized, double-blind comparative study of ADL-PF and ADL-EU in patients with active RA (REFLECTIONS B538–02), the treatment difference in the primary endpoint (ACR criteria for ≥ 20% clinical improvement [ACR20] at week 12) was within the pre-specified margins for therapeutic equivalence. In addition, there were no clinically meaningful differences in safety, immunogenicity, PK, or pharmacodynamics (PD) [14]. Together, the data demonstrated that ADL-PF fulfilled the requirements for biosimilarity to reference ADL, as per the guidance from the US Food and Drug Administration (FDA) and European Medicines Agency (EMA), with respect to structure, function, toxicity, PK and PD, clinical immunogenicity, and clinical safety and effectiveness [10, 11]. In the same comparative study of ADL-PF and ADL-EU in patients with active RA, comparable efficacy, safety, and immunogenicity between ADL-PF and ADL-EU was maintained up to week 56 and was unaffected by a blinded switch from ADL-EU to ADL-PF at week 26 [15].

Obtaining clinical evidence concerning switching of patients from boDMARDs to bsDMARDs, as well as following longer-term treatment, is key to instilling patient and clinician confidence. Here, we report findings from REFLECTIONS B538–02 on the long-term safety, immunogenicity, and efficacy of ADL-PF in patients who continued ADL-PF treatment throughout 78 weeks, or who switched from ADL-EU to ADL-PF at week 26 or week 52.

## Methods

The methodology for this comparative study has been described previously and is summarized briefly here [14].

### Study population

Inclusion and exclusion criteria have been described in detail elsewhere [14]. Eligible patients were adults ≥ 18 years, diagnosed with active RA for at least 4 months based on the 2010 American College of Rheumatology/European League Against Rheumatism (ACR/EULAR) criteria [16] and had an inadequate response to methotrexate (MTX). Active RA was defined as ≥ 6 tender and swollen joints at screening and baseline, and high-sensitivity C-reactive protein (hs-CRP) of ≥ 8 mg/L. Patients were required to have been treated with MTX for at least 12 weeks and to have been on a stable dose of MTX of 10–25 mg/week for ≥ 4 weeks with the exception of 6 to 25 mg/week where 6 mg/week was a recommended initial dose by local guidance or standard of care. Patients were excluded if they had previously received treatment with reference ADL, a lymphocyte depleting therapy, or more than two doses of one biologic therapy.

### Study design and treatments

**Fig. 1:**
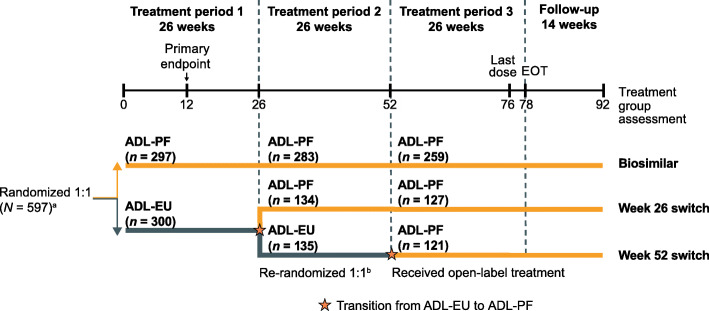
Study design. ^a^Randomization stratified by geographic regions (North America and Western Europe; Japan; Republic of Korea and Taiwan; Latin America; rest of world). ^b^In a blinded manner. ADL-EU, reference adalimumab sourced from the European Union; ADL-PF, PF-06410293; EOT, end of treatment

This was a multinational, two-arm, randomized, double-blind, parallel-group, 78-week study, comprising three 26-week treatment periods and a 14-week follow-up period (Fig. 1). In treatment period (TP) 1, patients stratified by geographic regions were randomized (1:1) to receive ADL-PF or ADL-EU for 26 weeks. At the start of TP2 (week 26), patients receiving ADL-EU were blindly re-randomized (1:1) to remain on ADL-EU or transition to ADL-PF for 26 weeks. At the start of TP3 (week 52), patients received open-label treatment with ADL-PF for 26 weeks with the last dose of study drug administered at week 76 and an end of treatment visit at week 78. Patients were followed for 16 weeks after the last treatment dose (follow-up period to week 92). ADL-PF or ADL-EU was administered as a subcutaneous injection (40 mg every other week), in addition to a stable background dose of oral or intramuscular MTX (10–25 mg/week) with lower doses of MTX (6 mg/week) permitted if indicated in local guidance or standards of care.

### Endpoints and assessments

The primary endpoint of the clinical study (the proportion of patients achieving ACR20 response at week 12) has been reported elsewhere [14]. We report herein secondary efficacy endpoints of the study that were assessed in TP3 at weeks 52, 56, 66, 76, and 78, including the proportion of patients who achieved ACR20, ACR50, and ACR70; the Disease Activity Score in 28 joints: four components based on hs-CRP (DAS28-4CRP); and the mean change from study baseline (week 0) on the DAS28-4CRP, EULAR response, and DAS28-4CRP < 2.6 and ACR/EULAR remission.

Safety endpoints evaluated throughout TP3 and follow-up period included type, incidence, severity, seriousness, and investigator-determined relatedness of adverse events (AEs) and laboratory abnormalities. AEs were classified using the MedDRA (version 20.1) classification system. The severity of AEs was graded according to the National Cancer Institute Common Terminology Criteria for Adverse Events (version 4.03). Treatment-emergent AEs (TEAEs) were defined as those with onset on or after the first dose of study drug during TP3 as well as ongoing AEs at the start of TP3 that worsened on or after the first dose of study drug in TP3.

Immunogenicity endpoints, assessed as a secondary objective of the study, were the number and percentage of patients in TP3 and follow-up period who had positive anti-drug antibodies (ADAs), positive neutralizing antibodies (NAbs) (analyzed in ADA-positive samples only), ADA and NAb titers, and incidence of transient ADA response.^1^ Among the patients who newly developed ADAs during TP2, none tested positive for NAb. Therefore, no new efficacy or safety analyses were performed for the NAb-positive patient subgroup in TP2 or TP3.

PK serum samples were analyzed for ADL-PF and ADL-EU using a validated analytical assay QPS, LLC (Newark, Delaware, USA), with a lower limit of quantification of 250 ng/mL. The PD endpoint was serum hs-CRP concentration.

### Statistical methods

Three groups were evaluated corresponding to the treatment sequence during the study: biosimilar group; week 26 switch group; and week 52 switch group (Fig. 1). Efficacy results from TP3 (week 52–78) and safety and immunogenicity results from TP3 and follow-up period (week 52–92) were summarized using descriptive statistics. Efficacy endpoints were evaluated in the intent-to-treat (ITT) population, defined as all patients enrolled in TP3, without imputation for missing data. Safety and immunogenicity endpoints were evaluated in the TP3 safety population which included all patients enrolled and treated with ≥ 1 dose of study treatment in TP3. PK analysis was performed using the PK population, which was defined as all patients who received treatment in TP3 and who provided ≥ 1 post-drug concentration measurement. For the PK analysis, drug concentration–time data were summarized according to visit and treatment group. Serum drug concentrations were also summarized by ADA and NAb status. PD analysis using hs-CRP concentration over time was summarized by treatment group.

## Results

### Patient disposition and demographics

**Fig. 2:**
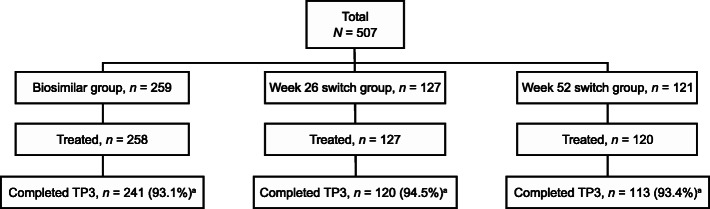
Patient disposition through TP3 (ITT population). ^a^Subjects who completed study treatment and had week-78 joint count assessment. ITT, intent-to-treat; TP3, treatment period 3

As previously reported, 597 patients were initially randomized to ADL-PF (*n* = 297) or ADL-EU (*n* = 300) in TP1 [14]. A total of 552 of 597 patients randomized in TP1 entered TP2 at week 26. A total of 507 patients continued in TP3 at week 56, comprising 259 in the biosimilar group, 127 in the week 26 switch group, and 121 in the week 52 switch group. Overall, 474 (93.5%) patients completed TP3, 93.1%, 94.5%, and 93.4% in the biosimilar, week 26 switch, and week 52 switch groups, respectively. Patient disposition through TP3 is shown in Fig. 2. There were no notable differences observed in baseline demographics and RA characteristics between the three treatment groups in TP3 (Table 1). Most patients were female (78.1%) and White (86.6%), and the average age was 52.1 years.

**Table 1:** Baseline demographics and clinical characteristics (ITT population: TP3)

	Biosimilar (***n*** = 259)	Week 26 switch (***n*** = 127)	Week 52 switch (***n*** = 121)	Total (***N*** = 507)
Female, *n* (%)	210 (81.1)	88 (69.3)	98 (81.0)	396 (78.1)
Age, years	51.1 (13.8)	53.1 (13.5)	53.1 (11.7)	52.1 (13.3)
Weight, kg	74.9 (18.0)	74.6 (17.7)	77.2 (21.2)	75.4 (18.7)
BMI, kg/m^2^	27.6 (6.3)	27.1 (5.8)	28.8 (7.6)	27.8 (6.5)
Race, *n* (%)				
White	230 (88.8)	109 (85.8)	100 (82.6)	439 (86.6)
Black	5 (1.9)	2 (1.6)	7 (5.8)	14 (2.8)
Asian	11 (4.2)	6 (4.7)	7 (5.8)	24 (4.7)
Other	13 (5.0)	10 (7.9)	7 (5.8)	30 (5.9)
Ethnicity, *n* (%)				
Not Hispanic/Latino	236 (91.1)	117 (92.1)	108 (89.3)	461 (90.9)
RA duration, years	6.8 (7.2)	6.5 (7.0)	7.0 (6.6)	6.8 (7.0)
RF or anti-CCP antibody positive, *n* (%)	208 (80.3)	102 (80.3)	105 (86.8)	415 (81.9)
Swollen joint count	15.0 (7.6)	16.8 (10.1)	17.3 (9.8)	16.0 (8.9)
Tender joint count	23.8 (11.9)	25.4 (14.8)	26.5 (15.0)	24.9 (13.5)
hs-CRP, mg/L	20.4 (20.4)	22.7 (26.5)	22.3 (25.3)	21.4 (23.3)
MTX dose, mg/week	15.3 (4.4)	14.6 (4.1)	15.8 (4.6)	15.2 (4.4)
Corticosteroid use, *n* (%)	145 (56.0)	79 (62.2)	68 (56.2)	292 (57.6)

Data are mean (SD) unless otherwise stated

*BMI* body mass index, *CCP* cyclic citrullinated peptides, *hs-CRP* high-sensitivity C-reactive protein, *ITT* intent-to-treat, *MTX* methotrexate, *RA* rheumatoid arthritis, *RF* rheumatoid factor, *SD* standard deviation, *TP3* treatment period 3

### Efficacy

**Fig. 3:**
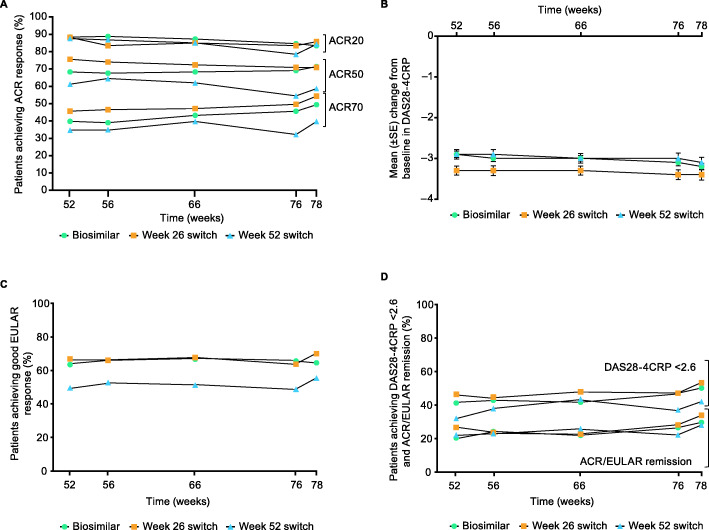
**A** Proportion of patients achieving ACR response, **B** mean (± SE) change from study baseline in DAS28-4CRP, **C** proportion of patients achieving good EULAR response, and **D** proportion of patients achieving DAS-4CRP < 2.6 and ACR/EULAR remission (ITT population in TP3). ACR20/50/70, ACR criteria for ≥ 20%/50%/70% clinical improvement; DAS28-4CRP, Disease Activity Score in 28 joints: 4 components based on high-sensitivity C-reactive protein; ITT, intent-to-treat; SE, standard error; TP3, treatment period 3

As previously reported, therapeutic equivalence of ADL-PF and ADL-EU was demonstrated based on the two-sided 95% confidence interval (CI) for the treatment difference in the primary endpoint (week-12 ACR20) being entirely contained within ± 14% symmetric margin, as well as by the two-sided 90% CI being entirely contained within asymmetric margin of − 12 to 15% as specified by the FDA. ACR20, ACR50, and ACR70 response rates for all patients were 88.2%, 68.4%, and 40.0%, respectively, at week 52, and 84.2%, 68.2%, and 48.3%, respectively, at 78 weeks. For the biosimilar, week 26 switch, and week 52 switch groups, the ACR20 response rates prior to the first injection of study drug (week 52) were 88.4%, 88.2%, and 87.6%, respectively, and were 83.4%, 85.8%, and 84.3%, respectively, at week 78 (Fig. 3A). ACR20 response rates were sustained and comparable across the three groups at all TP3 visits. ACR50 and ACR70 response rates were numerically lower in the week 52 switch group compared with the other two groups and were overall generally sustained through the week 78 visit in all groups (Fig. 3A).

Mean DAS28-4CRP score for all patients was 3.0 at week 52 reflecting a mean change from study baseline of − 3.0 and at week 78 was 2.8 reflecting a mean change from study baseline of − 3.2. The mean DAS28-4CRP scores and the mean change from study baseline on DAS28-4CRP were comparable between the three treatment groups at all TP3 visits (Fig. 3B).

Overall, a good EULAR response was reached by 61.9% of patients at week 52 and by 63.7% of patients at week 78. Overall, the EULAR response rates at week 52 persisted in TP3 across the three treatment groups (Fig. 3C). Numerically lower rates of good EULAR response were observed in patients in the week 52 switch group compared with the other two groups and were overall generally sustained through the week 78 visit in all groups. DAS28-4CRP < 2.6 and remission based on the ACR/EULAR criteria was achieved by 40.4% and 22.5%, respectively, of all patients at week 52 and by 49.1% and 30.4%, respectively, at week 78. DAS28-4CRP < 2.6 and ACR/EULAR remission rates were sustained and comparable across the three treatment groups in TP3 (Fig. 3D).

### Safety

A total of 218 (43.2%) patients reported 446 all-causality TEAEs during TP3 and follow-up period; 21 (4.2%) patients reported serious AEs (SAEs) and 28 (5.5%) patients reported grade 3 or higher TEAEs. Eleven (2.2%) patients discontinued treatment due to TEAEs in TP3 (Table 2). TEAEs occurring in ≥ 2% of patients overall were RA (5.7%), nasopharyngitis (5.1%), hypertension (2.2%), anemia (2.0%), and upper respiratory tract infection (2.0%) (Table 2). A total of 57 (11.3%) patients reported 77 treatment-related TEAEs in TP3 and the follow-up period, with infections and infestations being the most commonly reported class of treatment-related TEAEs (27 patients; 5.3%). Three (0.6%) patients reported treatment-related SAEs. There was one death in the study that occurred in the week 52 switch group due to gastrointestinal bleed, aspiration pneumonia, and septic shock. Concerning TEAEs of special interest, injection-site reactions were reported in two patients in the biosimilar group and there was one case of latent tuberculosis in this group. There were two reported cases of urticaria in the week 26 switch group. A single case of septic shock was reported in the week 52 switch group.

**Table 2:** All-cause TEAEs during TP3 and follow-up (safety population; TP3)

	Biosimilar (***n*** = 258)	Week 26 switch (***n*** = 127)	Week 52 switch (***n*** = 120)	Total (***N*** = 505)
Number of AEs	206	94	146	446
Patients with events				
AEs	110 (42.6)	47 (37.0)	61 (50.8)	218 (43.2)
SAEs	9 (3.5)	3 (2.4)	9 (7.5)	21 (4.2)
Grade 3 AEs	14 (5.4)	3 (2.4)	7 (5.8)	24 (4.8)
Grade 4 AEs	0	0	3 (2.5)	3 (0.6)
Grade 5 AEs	0	0	1 (0.8)	1 (0.2)
Patients who discontinued due to AEs				
From treatment temporarily	14 (5.4)	2 (1.6)	9 (7.5)	25 (5.0)
From treatment permanently	6 (2.3)	2 (1.6)	3 (2.5)	11 (2.2)
From the study	4 (1.6)	1 (0.8)	3 (2.5)	8 (1.6)
AEs occurring in ≥ 2% of patients in any treatment group				
Anemia	5 (1.9)	1 (0.8)	4 (3.3)	10 (2.0)
Neutropenia	1 (0.4)	3 (2.4)	1 (0.8)	5 (1.0)
Diarrhea	3 (1.2)	2 (1.6)	3 (2.5)	8 (1.6)
Bronchitis	5 (1.9)	1 (0.8)	3 (2.5)	9 (1.8)
Nasopharyngitis	13 (5.0)	10 (7.9)	3 (2.5)	26 (5.1)
Influenza	4 (1.6)	1 (0.8)	3 (2.5)	8 (1.6)
Upper respiratory tract infection	2 (0.8)	6 (4.7)	2 (1.7)	10 (2.0)
Urinary tract infection	2 (0.8)	3 (2.4)	2 (1.7)	7 (1.4)
ALT increased	3 (1.2)	3 (2.4)	3 (2.5)	9 (1.8)
AST increased	3 (1.2)	1 (0.8)	3 (2.5)	7 (1.4)
Back pain	2 (0.8)	2 (1.6)	3 (2.5)	7 (1.4)
Rheumatoid arthritis	13 (5.0)	5 (3.9)	11 (9.2)	29 (5.7)
Headache	6 (2.3)	0	3 (2.5)	9 (1.8)
Hypertension	4 (1.6)	5 (3.9)	2 (1.7)	11 (2.2)

Data are presented as *n* (%), unless otherwise indicated

An AE is considered a TEAE for TP3 if it has an onset date on or after the date of the first dose of study drug (week 52 dosing) or is a baseline event that increases in severity during TP3

*AE* adverse event, *ALT* alanine aminotransferase, *AST* aspartate aminotransferase, *SAE* serious adverse event, *TEAE* treatment-emergent adverse event, *TP3* treatment period 3

### Immunogenicity

**Fig. 4:**
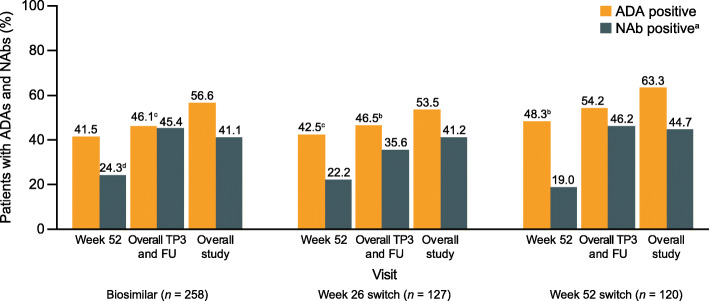
The proportions of patients who tested positive for ADAs and, of those, the proportions who tested positive for NAbs, by study visit (safety population; TP3 and follow-up). ^a^NAb-positive incidences are expressed as percentage of ADA-positive patients; not tested for ADAs. ^b^*n* = 1. ^c^*n* = 2; not tested for NAbs. ^d^*n* = 1. ADA, anti-drug antibody; FU, follow-up; NAb, neutralizing antibody; TP3 treatment period 3

The percentages of patients with ADA overall during TP3 and the follow-up period were 46.1%, 46.5%, and 54.2% for the biosimilar, week 26 switch, and week 52 switch groups, respectively. Of these patients, 45.4% (biosimilar), 35.6% (week 26 switch), and 46.2% (week 52 switch) also tested positive for NAb. The incidences of patients with ADAs and NAbs at pre-dose (TP3) week 52, overall during TP3 and the follow-up period, and overall during the study were comparable between the three groups (Fig. 4).

The incidences of patients with a first positive ADA result during TP3 or follow-up, among those who were ADA negative on entry to TP3, were comparable between the three treatment groups at 8.9%, 6.3%, and 8.3% for the biosimilar, week 26, and week 52 switch groups, respectively. The distribution of ADA titers was comparable across the three treatment groups and generally stable over time. Transient ADA responses (i.e., treatment-induced ADAs detected at only a single visit or ADAs detected at ≥ 2 visits where the first and last ADA-positive samples were separated by < 16 weeks, with an ADA-negative result at the last sampling time-point for both cases) were determined based on data from all three treatment periods and the follow-up period. Transient ADA response in the ADA-positive population was comparable in the biosimilar (15.8%), week 26 switch (10.3%), and week 52 switch (10.5%) groups. Among the patients who developed ADAs overall during the study, 10/290 (3.4%) had a hypersensitivity TEAE (Table S1) and 1/290 (0.3%) had an injection-site reaction on or after the date of a first ADA-positive test.

### Pharmacokinetics

**Fig. 5:**
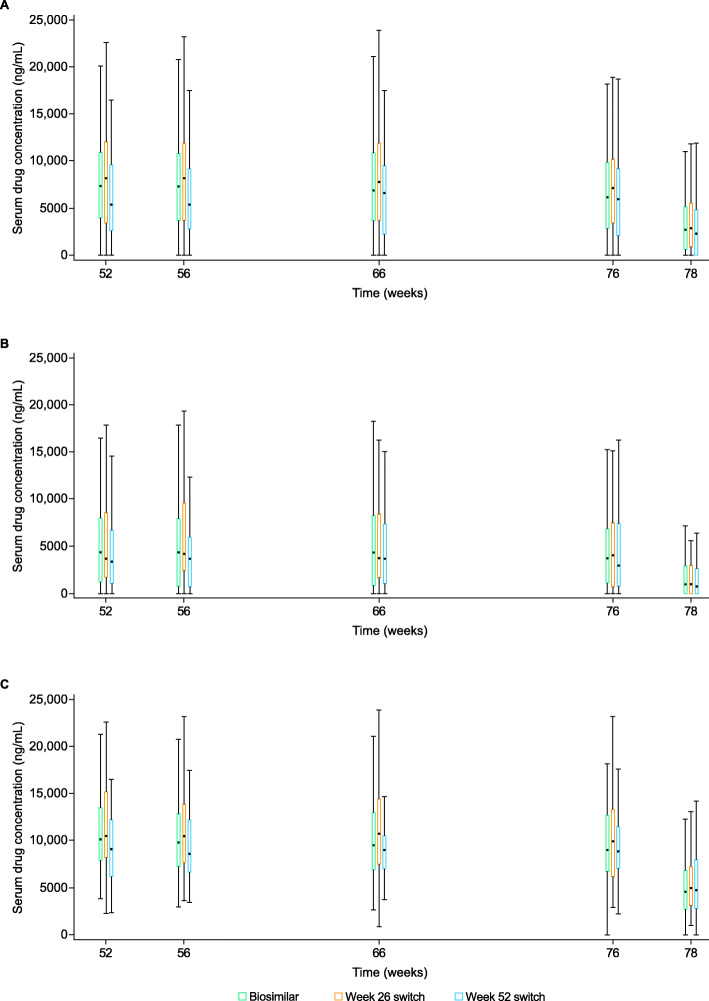
Serum drug concentration–time profile for biosimilar, week 26 switch, and week 52 switch treatment groups **A** overall, **B** in ADA-positive patients, and **C** in ADA-negative patients. Summary statistics were calculated by setting concentration values below LLOQ to 0 (LLOQ = 250 ng/mL). Box plot provides median and 25%/75% quartiles with whiskers to the last point within 1.5 × the interquartile range. Unplanned readings were excluded from the presentation. Stars, circles, and squares represent median values. TP3 included data from week 56, week 66, EOT/ET, and follow-up visits. Data from week 52 represented samples obtained in TP2 prior to dosing and are included here for visualization purposes. ADA, anti-drug antibody; EOT, end of treatment; ET, early termination; LLOQ, lower limit of quantification; PK, pharmacokinetic; TP2, treatment period 2; TP3, treatment period 3

Mean serum drug concentrations were generally comparable across all three treatment groups at each TP3 visit and during the follow-up period (Fig. 5A). As expected, mean serum drug trough concentrations for ADA-positive patients were lower compared with ADA-negative patients in all treatment groups (Fig. 5B, C). At week 78, mean serum drug trough concentrations for ADA-positive and ADA-negative patients, respectively, were 4529 and 9584 ng/mL (biosimilar group), 4725 and 10,470 ng/mL (week 26 switch group), and 4197 and 9728 ng/mL (week 52 switch group). Mean serum drug trough concentrations were lower for ADA-positive/NAb-positive patients compared with ADA-positive/NAb-negative patients at each TP3 visit and during follow-up (Figure S1). At week 78, mean serum drug trough concentrations were 1516 ng/mL (biosimilar), 2070 ng/mL (week 26 switch), and 1042 ng/mL (week 52 switch) for ADA-positive/NAb-positive patients and were 6624 ng/mL (biosimilar), 6562 ng/mL (week 26 switch), and 6800 ng/mL (week 52 switch) for ADA-positive/NAb-negative patients.

### Pharmacodynamics

Mean hs-CRP concentrations at week 52 pre-dose were 9.7, 9.8, and 10.4 mg/L in the biosimilar, week 26 switch, and week 52 switch groups, respectively, and at week 78 were 10.5, 8.2, and 9.4 mg/L, respectively. The mean change from study baseline in hs-CRP concentrations were − 10.7, − 12.9, and − 11.9 mg/L at week 52 pre-dose, and − 9.7, − 13.3, and − 11.9 mg/L at week 52 in the biosimilar, week 26 switch, and week 52 switch groups, respectively.

## Discussion

Acquiring data on switching and longer-term efficacy and safety of biosimilars in the clinical setting, beyond the data required for support of regulatory approval, is valuable in informing payor and clinician decision-making. Here, we report data from TP3 and the long-term safety follow-up period of REFLECTIONS B538–02, a double-blind, comparative study of ADL-PF and ADL-EU in patients with active RA. Therapeutic equivalence between the ADL-PF (a bsDMARD) and ADL-EU (the boDMARD) was established based on the primary endpoint of ACR20 response at week 12 [14]. The efficacy, safety, and immunogenicity data from this study contributed to the totality of the evidence supporting regulatory approval by the EMA and FDA of ADL-PF for all eligible indications for which reference ADL is licensed.

During TP3 (from week 52 to 78), all patients received open-label treatment with ADL-PF. The efficacy of ADL-PF, as determined by the ACR response rates, was comparable between the three treatment groups during TP3, which were based on the treatment sequence the patients received during the study (Fig. 3A). ACR50 and ACR70 response rates were numerically lower in the week 52 switch group compared with the other two groups during TP3; however, there were no clinically meaningful differences between patients maintained on ADL-PF for 78 weeks (biosimilar group) and those who were switched from ADL-EU either at week 26 (week 26 switch group) or week 52 (week 52 switch group). Generally comparable responses across groups were also apparent from the assessment of other secondary efficacy outcome measures, such as change in DAS28-4CRP score from baseline, and the proportions of patients achieving a good EULAR response, DAS28-4CRP < 2.6, and ACR/EULAR remission. Numerically lower good EULAR response rates were observed in the week 52 switch group compared with the other two groups. The ACR20 response rates for patients who switched to ADL-PF from ADL-EU were also largely unaltered from the time of last treatment with ADL-EU to the end of the study (week 26 switch group, 86.6% [week 26] [15] and 85.5% [week 78]; week 52 switch group, 87.6% [week 52] and 84.3% [week 78]). For those patients receiving ADL-PF during the double-blind phase (TP1 and/or TP2) before entry into TP3 (i.e., biosimilar and week 26 switch groups), ACR20 response rates were maintained from the end of the double-blind phase (week 52) through the open-label phase to the end of study (week 78). Measures of the secondary efficacy outcomes were consistent with these ACR20 response rate profiles.

ADL-PF was generally well tolerated during TP3 and the safety profile of ADL-PF was consistent with the long-term profile established for ADL in patients with RA [17]. There were no clinically meaningful differences for the incidences and types of all-causality TEAEs across the three treatment groups. The majority of TEAEs were grade 1 or 2 and the frequency of events by severity grade was comparable among the treatment groups. The most frequently reported TEAEs were RA, nasopharyngitis, and hypertension. Given that the data for TP3 was analyzed using descriptive statistics, no inference was made for numerical differences between groups. The incidences of patients who discontinued treatment permanently due to AEs during TP3 were comparable between groups. Fewer than 2% of patients in any group reported pre-specified AEs of special interest, with no clinically meaningful differences in their reporting, regardless of causality. The incidence of both ADAs and NAb development among the ADA-positive patients was comparable between treatment groups. There were no clinically meaningful differences in the proportions of patients who tested positive for ADAs and who showed NAb positivity. Limitations of the study include the absence of patients maintained on ADL-EU throughout as a control group.

## Conclusions

Results from TP3 of this study, in which all patients received open-label treatment with ADL-PF, showed that there were no clinically meaningful differences in safety, immunogenicity, or efficacy between patients who had been maintained on ADL-PF for 78 weeks and those who had switched from ADL-EU at week 26 or week 52. These data, demonstrating response maintenance after switching, together with results from the previously reported primary analysis establishing therapeutic equivalence of ADL-PF with ADL-EU at week 12, support the similarity of ADL-PF to reference ADL.

## Supplementary Information

**Additional file 1: **Figure S1 Serum drug concentration–time profile for ADA-positive patients in the biosimilar, week 26 switch, and week 52 switch treatment groups, by neutralizing antibody status during TP3 **A** NAb positive, **B** NAb negative.

**Additional file 2:** Table S1. Hypersensitivity TEAEs (PT) on or after the date of subject first ADA-positive test (safety population; TP3).

## Data Availability

Upon request, and subject to certain criteria, conditions, and exceptions (see https://www.pfizer.com/science/clinical-trials/trial-data-and-results for more information), Pfizer will provide access to individual de-identified participant data from Pfizer-sponsored global interventional clinical studies conducted for medicines, vaccines and medical devices (1) for indications that have been approved in the US and/or EU or (2) in programs that have been terminated (i.e., development for all indications has been discontinued). Pfizer will also consider requests for the protocol, data dictionary, and statistical analysis plan. Data may be requested from Pfizer trials 24 months after study completion. The de-identified participant data will be made available to researchers whose proposals meet the research criteria and other conditions, and for which an exception does not apply, via a secure portal. To gain access, data requestors must enter into a data access agreement with Pfizer.

## Abbreviations

ACR: American College of Rheumatology
ACR20/50/70: ACR criteria for ≥ 20%/50%/70% clinical improvement
ADA: Anti-drug antibody
ADL: Adalimumab
ADL-EU: Reference adalimumab sourced from the European Union
ADL-PF: PF-06410293
ADL-US: Reference adalimumab sourced from the United States
AE: Adverse event
ALT: Alanine aminotransferase
AST: Aspartate aminotransferase
BMI: Body mass index
boDMARD: Biologic original disease-modifying anti-rheumatic drug
bsDMARD: Biosimilar disease-modifying anti-rheumatic drug
CI: Confidence interval
DAS28-4CRP: Disease Activity Score in 28 joints, 4 components based on high-sensitivity C-reactive protein
DMARD: Disease-modifying anti-rheumatic drug
EMA: European Medicines Agency
EOT: End of treatment
ET: Early termination
EULAR: European League Against Rheumatism
FDA: Food and Drug Administration
FU: Follow-up
hs-CRP: High-sensitivity C-reactive protein
ITT: Intent-to-treat
LLOQ: Lower limit of quantification
MTX: Methotrexate
NAb: Neutralizing antibody
PD: Pharmacodynamics
PK: Pharmacokinetic
RA: Rheumatoid arthritis
SAE: Serious adverse event
SD: Standard deviation
SE: Standard error
TEAE: Treatment-emergent adverse event
TNF-α: Tumor necrosis factor-α
TP: Treatment period

## Footnotes

1 A transient ADA response was defined as having a treatment-induced ADA detected at only one sampling time-point during treatment or follow-up, or by treatment-induced ADA detected at ≥ 2 sampling time-points during treatment or follow-up, where the first and last ADA-positive samples were separated by a period < 16 weeks, and the subject’s last sampling time-point result was ADA-negative.
