# Supplementary material for: Long-term efficacy, safety, and immunogenicity of the adalimumab biosimilar, PF-06410293, in patients with rheumatoid arthritis after switching from reference adalimumab (Humira®) or continuing biosimilar therapy: week 52–92 data from a randomized, double-blind, phase 3 trial
Source: Arthritis Res Ther. 2021 Sep 25;23:248. doi: 10.1186/s13075-021-02626-4 (PMC8464121; doi:10.1186/s13075-021-02626-4)
Supplement: Supplementary file 1 — Additional file 1: Figure S1 Serum drug concentration–time profile for ADA-positive patients in the biosimilar, week 26 switch, and week 52 switch treatment groups, by neutralizing antibody status during TP3 A NAb positive, B NAb negative. [file 13075_2021_2626_MOESM1_ESM.docx]

**Figure S1** Serum drug concentration–time profile for ADA-positive patients in the biosimilar, week 26 switch, and week 52 switch treatment groups, by neutralizing antibody status during TP3 **A** NAb positive, **B** NAb negative


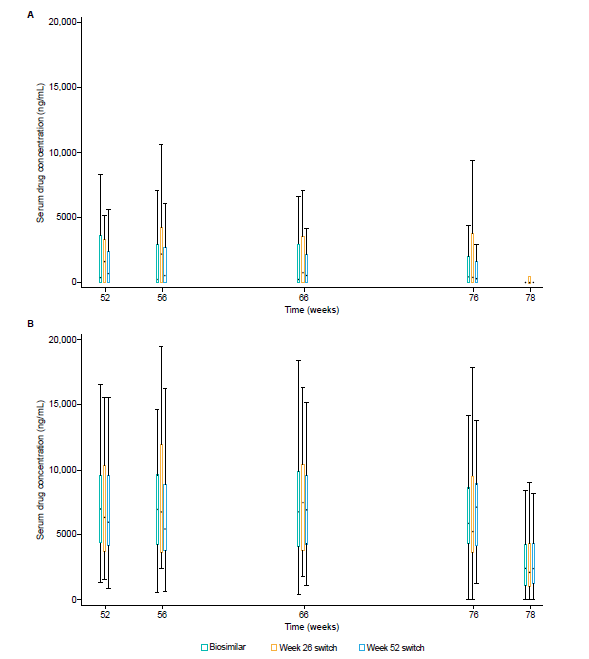


Summary statistics were calculated by setting concentration values below the lower limit of quantification to zero. Stars, circles, and squares represent median values. Box plot provides median and 25%/75% quartiles with whiskers to the last point within 1.5 × the interquartile range. Unplanned readings have been excluded from the presentation. TP3 includes data from week 56, week 66, EOT/ET, and follow-up visits. Data from week 52 represent samples obtained in TP2 prior to dosing and are included here for visualization purposes.

*ADA* anti-drug antibody, *EOT* end of treatment, *ET* early termination, *NAb* neutralizing antibody, *TP2* treatment period 2, *TP3* treatment period 3
