# Supplementary material for: Long-term efficacy, safety, and immunogenicity of the adalimumab biosimilar, PF-06410293, in patients with rheumatoid arthritis after switching from reference adalimumab (Humira®) or continuing biosimilar therapy: week 52–92 data from a randomized, double-blind, phase 3 trial
Source: Arthritis Res Ther. 2021 Sep 25;23:248. doi: 10.1186/s13075-021-02626-4 (PMC8464121; doi:10.1186/s13075-021-02626-4)
Supplement: Supplementary file 2 — Additional file 2: Table S1. Hypersensitivity TEAEs (PT) on or after the date of subject first ADA-positive test (safety population; TP3). [file 13075_2021_2626_MOESM2_ESM.docx]

**Table S1.** Hypersensitivity TEAEs (PT) on or after the date of subject first ADA-positive test (safety population; TP3)

| **Number of patients evaluable for AEs** | **Biosimilar (*n* = 146)** | **Week 26 switch (*n* = 68)** | **Week 52 switch  (*n* = 76)** | **Total**  **(*N* = 290)** |
| --- | --- | --- | --- | --- |
| Patients with AEs | 4 (2.7) | 3 (4.4) | 3 (3.9) | 10 (3.4) |
| Cough | 2 (1.4) | 0 | 0 | 2 (0.7) |
| Double stranded DNA antibody-positive | 0 | 1 (1.5) | 0 | 1 (0.3) |
| Eczema | 1 (0.7) | 0 | 0 | 1 (0.3) |
| Erythema | 0 | 0 | 1 (1.3) | 1 (0.3) |
| Rash | 1 (0.7) | 0 | 0 | 1 (0.3) |
| Rash macular | 1 (0.7) | 0 | 0 | 1 (0.3) |
| Respiratory failure | 0 | 0 | 1 (1.3) | 1 (0.3) |
| Seasonal allergy | 0 | 0 | 1 (1.3) | 1 (0.3) |
| Shock | 0 | 0 | 1 (1.3) | 1 (0.3) |
| Urticaria | 0 | 2 (2.9) | 0 | 2 (0.7) |

Data are presented as *n* (%)

*ADA* anti-drug antibody, *AE* adverse event, *PT* preferred term, *TEAE* treatment-emergent adverse event, *TP3* treatment period 3
